# Iron, Meat and Health 

**DOI:** 10.3390/nu3030283

**Published:** 2011-02-28

**Authors:** Catherine Geissler, Mamta Singh

**Affiliations:** 1 Nutritional Sciences Division, King’s College London and MRC Human Nutrition Research, Elsie Widdowson Laboratory, Cambridge CB1 9NL, UK; 2 Department of Health, 133-155 Waterloo Road, London, SE1 8UG, UK; Email: Mamta.Singh@dh.gsi.gov.uk

**Keywords:** iron, diet, health

## Abstract

This article is a summary of the publication “Iron and Health” by the Scientific Advisory Committee on Nutrition (SACN) to the U.K. Government (2010), which reviews the dietary intake of iron and the impact of different dietary patterns on the nutritional and health status of the U.K. population. It concludes that several uncertainties make it difficult to determine dose-response relationships or to confidently characterize the risks associated with iron deficiency or excess. The publication makes several recommendations concerning iron intakes from food, including meat, and from supplements, as well as recommendations for further research.

## 1. Introduction

Iron deficiency and iron deficiency anemia are serious public health problems throughout the life cycle in both industrialized and developing countries, with negative consequences on maternal and child mortality, cognitive and physical development of children, and physical performance and work capacity in adults. Strategies to control deficiencies include dietary diversification, food fortification and iron supplementation. However there are concerns that increased iron intakes from high meat consumption, and iron fortification and supplementation may lead to increased risk of chronic diseases including cardiovascular disease and cancer, especially in sections of populations with genetic predispositions to iron overload. 

This article is based on the recently published review of Iron and Health by the Scientific Advisory Committee on Nutrition (SACN) to the UK government [1]. The review was undertaken following the recommendation of the previous advisory committee, the Committee on the Medical Aspects of Food and Nutrition Policy (COMA) in their report, *Nutritional Aspects of the Development of Cancer* [2] which had concluded that lower consumption of red and processed meat would probably reduce the risk of colorectal cancer. COMA recommended that intakes of red and processed meat should not rise, and that adults with intakes greater than average (then estimated to be 90 g/day cooked weight), especially those with high intakes, should consider a reduction in intake. Since red meat is a source of iron (as well as other micronutrients such as zinc) in the UK diet, COMA recommended that the “*implications of a reduction in meat consumption on other aspects of health, particularly iron status*” should be reviewed.

However in view of the possibility that some subgroups of the U.K. population might be iron deficient, or be at risk of iron deficiency, and of possible adverse effects of increased iron intakes, consideration of iron nutrition in the U.K. population needs to be sensitive to the balance between iron deficiency and iron excess.

The terms of reference of the SACN Iron Working Group were:

To review the dietary intakes of iron in its various forms and the impact of different dietary patterns on the nutritional and health status of the population and to make proposals.

Consideration of both beneficial and adverse effects of increasing iron intakes was undertaken, including: the effect of dietary components on iron absorption and utilization in the body; interactions of infections and inflammation with iron metabolism and the possibility that this may affect the apparent incidence of iron deficiency; the effect of iron deficiency on health and well-being, for example mental and physical development; potential adverse effects of excess iron, including free radical damage and the risk of cardiovascular disease (CVD) and cancer; as well as the associations between consumption of red and processed meat and cancer risk since these foods contain iron. These are summarized here.

## 2. Biochemistry

Iron (Fe) exists in two biologically relevant states: the reduced ferrous form (Fe^2+^) and the oxidized ferric form (Fe^3+^). Iron is an efficient catalyst for electron transfer and free-radical reactions which also means that “free iron” (*i.e.*, when it is not bound to protein or other organic molecules) is potentially toxic and that organisms need to minimize their exposure to it. This protection from exposure depends on proteins which are specifically involved in its uptake from the diet and its transfer into the systemic circulation, its transport around the body and storage in tissues, as well as its delivery to functional sites.

## 3. Function

Iron, as a component of hemoglobin in erythrocytes (red blood cells), is required for transporting oxygen around the body and, in the form of myoglobin, for the storage and use of oxygen in muscles. The oxygen released in the tissues from hemoglobin is used in oxidative metabolism. Hemoglobin binds carbon dioxide in the tissues and carries it to the lungs where it is exhaled. 

**Table 1:** Examples of functional iron-containing proteins in the body (75 kg man).

Iron-containing protein	Function	Location	Iron content (mg)
**Heme proteins**			
Hemoglobin	Oxygen transport	Red blood cells	3,000
Myoglobin	Oxygen storage	Muscle	400
**Heme enzymes**		All tissues	c 30
Cytochromes a, b, c	Electron transfer		
	Transfer of electrons to molecular oxygen at end of respiratory chain (also requires copper)		
			
Cytochrome C oxidase	Microsomal mixed function oxidases		
Cytochrome P450 + b_5_	Phase I biotransformation of xenobiotics		
Dcytb	Ferrireductase (duodenal enterocytes)		
			
Catalase	Hydrogen peroxide breakdown		
Peroxidases	Peroxide breakdown		
Myeloperoxidase	Neutrophil bacteriocide		
			
Sulfite oxidase	Sulfites to sulfates		
			
Tryptophan 2,3-dioxygenase	Pyridine metabolism		
Iodase (iodoperoxidase)	Iodide to iodate		
			
**Non-heme iron enzymes**		All tissues	c 30
Ribonucleotide reductase	Ribonucleotides → 2’-deoxyribnucleotides		
	Synthetic phase of cell division		
***(Iron-sulfur proteins)***			
Aconitase	Citric acid cycle and initial steps of oxidative phosphorylation		
Isocitrate dehydrogenase			
Succinate dehydrogenase			
NADH dehydrogenase			
			
Aldehyde oxidase	Aldehydes to carboxylic acids		
Xanthine oxidase	Hypoxanthine-uric acid		
			
Phenylalanine hydroxylase	Catecholamine, neurotransmitters, and melanin synthesis		
Tyrosine hydroxylase			
Tryptophan hydroxylase			
			
Prolyl hydroxylase	Collagen synthesis, both dependent on ascorbic acid		
Lysyl hydroxylase			

Iron is also present as a component of iron-sulfur complexes in enzymes that are responsible for electron transport and energy generation in mitochondrial respiration and the citric acid cycle, and for ribonucleotide reductase, which is essential for DNA synthesis (Table 1). Body iron content is approximately 4.0 g in men and 3.5 g in women. In adults, most body iron is present in hemoglobin (60–70%) in circulating erythrocytes where it is essential for oxygen transport, and in muscle myoglobin (10%). The remaining body iron (20–30%) is found primarily in storage pools located in the liver and reticulo-endothelial (macrophage) system as ferritin and hemosiderin. Only about 1% of body iron is incorporated in the range of iron-containing enzymes and less than 0.2% of body iron is in the plasma transport pool where it is bound to transferrin.

## 4. Metabolism

Absorption of iron from the gastrointestinal tract is regulated by the systemic need for iron. A series of organic molecules have specific roles in binding free iron, carrying it in the circulation, and delivering it to functional sites or, if not immediately needed, depositing it in a safe form in ferritin. The principal pool of ferritin is in the liver which holds approximately 25% of body iron: two-thirds as ferritin and up to one-third as insoluble hemosiderin. The body has no means of excreting excess iron. The only way in which iron is lost from the body is from desquamated skin cells and sweat (0.2–0.3 mg/day), urine (<0.1 mg/day), gastrointestinal secretions and hair and, in premenopausal women, from menses. Erythrocytes have a life span of approximately 120 days and are then engulfed and destroyed by the macrophages of the reticulo-endothelial system which recycle approximately 30 mg/day of iron from senescent erythrocytes. In healthy individuals obligatory iron losses from the skin and gastrointestinal mucosa are thought to be approximately 1 mg/day in males [3] and slightly more in women of child-bearing age because of additional losses due to menstruation, pregnancy, and lactation.

## 5. Absorption

Iron uptake and transfer depends on specific cellular carrier mechanisms. The principal, and probably the only, physiological and primary determinant of how much iron is absorbed is the systemic need for iron*.* Absorption occurs mainly in the proximal small intestine and involves the uptake and transfer of iron across the enterocyte into the systemic circulation. Carrier proteins are situated on the apical surface of the enterocytes, which is in contact with the intestinal lumen and its contents, and at their basal surface which is in contact with the circulation. 

There are at least two separate mechanisms for the uptake of heme and non-heme iron into the enterocyte. The divalent metal transporter 1 (DMT1) transports inorganic iron and is specific for ferrous iron. Duodenal cytochrome B reductase (DcytB) converts dietary ferric iron to the more soluble ferrous state. In the enterocyte, ferrous iron enters a labile or “exchangeable” iron pool from which it can enter three different pathways, depending on the requirements of the body: taken into the local mitochondria for heme synthesis; sequestered into ferritin iron depots (and shed into the gut lumen at the end of the enterocyte’s lifespan); or transferred to the basal transporter (ferroportin 1) for translocation into the body. The mechanism of heme iron absorption remains unclear. The suggested role of the heme carrier protein 1 (HCP 1) in heme transport is now uncertain [4]. However, once in the enterocyte the heme molecule is degraded by heme oxygenase to release ferric iron [5] which then enters the enterocytic exchangeable pool.

An efficient pathway exists for the intestinal uptake of ferritin, derived from plant and meat based dietary sources, which involves lysosomal dissolution of the ferritin core to release the iron [6,7,8]. Hephaestin, a ferroxidase found mostly in the basal membrane of enterocytes, is thought to facilitate basolateral iron export from the intestinal epithelial cells by oxidizing the ferrous iron back to its ferric form, possibly with ferroportin 1 [9]. Ceruloplasmin, which is found in plasma, is also a ferroxidase and may be involved in the oxidation of ferrous iron to ferric iron during binding to transferrin.

Regulation of intestinal iron absorption occurs both at the stage of mucosal uptake and at the stage of its transfer to the blood. Changes in iron absorption are mediated by maturing enterocytes in the mucosal crypts and do not become effective until the newly matured enterocytes have moved to the villi, resulting in a time lag of one to two days between changes in systemic iron need and the corresponding mucosal setting for iron uptake and transfer. A large intake of dietary iron can, itself, induce the enterocytes to develop a “mucosal block” [10], reducing the intestinal transfer of iron for several days [11] even in the presence of systemic iron deficiency. 

The principal regulator of iron absorption is hepcidin [12], predominantly expressed in the liver. It down regulates iron absorption in the small intestine, the transport of iron across the placenta, and the release of iron from macrophages and hepatocytes [13]. The mechanism of hepcidin action may be twofold and cell-type specific: in macrophages, it binds to and degrades ferroportin on the cellular membrane, which prevents iron from leaving the cell [14], however, in enterocytes it may also down regulate iron uptake by inhibiting DMT1 transcription [15,16]. Hepatic hepcidin production is increased when iron stores are adequate or high and during inflammation. When systemic iron requirements are increased or iron stores are low, or both, hepcidin production is decreased. Hepcidin production is also reduced by systemic hypoxia, stimulating the production of erythropoietin which induces the synthesis of new red blood cells. Defective regulation of hepcidin, or its receptor ferroportin, causes a range of iron overload disorders known as the hemochromatoses [17] (see Table 2), characterized by increased iron absorption, which leads to excessive systemic iron accumulation and overload. The most common form is associated with hepcidin deficiency. Hepcidin deficiency is a characteristic of mutations in the HAMP (hepcidin antimicrobial peptide) gene but most patients with genetic hemochromatosis have alterations in the HFE (high iron Fe) gene, or, rarely in the transferrin receptor 2 (TFR2) or hemojuvelin (HJV) genes, suggesting that these proteins are involved in the regulation of hepcidin synthesis [18].

## 6. Plasma Iron Transport

Iron is distributed around the body in the circulation as transferrin. Transferrin comprises a core carrier glycoprotein, apotransferrin, which can bind one or two atoms of ferric iron to form holotransferrin, which is more usually referred to as transferrin. The uptake of iron by cells is mediated by the binding of holotransferrin (Tf) to transferrin receptors (TfR) on the cell surface which is then internalized by endocytosis. The iron is then either stored as ferritin or used within the cell, for example for hemoglobin synthesis. The apotransferrin and the TfR return to the cell surface and the apotransferrin is recycled into the plasma. A second transferrin receptor (TFR2) is thought to be involved in the regulation of iron absorption by influencing hepcidin expression. Cells may also acquire iron through transferrin-independent pathways. The transmembrane protein, “*Stimulator of Fe Transport*”, facilitates the uptake of both ferrous and ferric iron independently of transferrin and may also have a role in intracellular iron transport [19,20,21]. Its significance in iron metabolism is presently unclear.

## 7. Iron Storage

The liver is a major systemic depot of iron. Iron is taken up by hepatocytes from transferrin and released in times of increased need, subject to regulation by hepcidin. In diseases which cause increased transferrin iron saturation and iron overload (see Table 2) the liver continues to accumulate iron, even when iron stores are high, and is therefore vulnerable to developing damage secondary to iron overload. 

**Table 2:** Classification of genetic hemochromatoses.

Type	Mutated protein	Mode of transmission	Phenotype	Mechanism	Severity	Relative incidence in populations of European origin
1	HFE	Recessive	Parenchymal iron overload	Hepcidin deficiency	Highly variable	Common (1 in 100–1 in 1000)
2A Juvenile hemochromatosis	Hemojuvelin	Recessive	Parenchymal iron overload. Early onset (2nd or 3rd decades)	Hepcidin deficiency	Severe	Rare
2B Juvenile hemochromatosis	Hepcidin	Recessive	Parenchymal iron overload. Early onset (2nd or 3rd decades)	Hepcidin deficiency	Severe	Rare
3	Transferrin receptor 2	Recessive	Parenchymal iron overload	Hepcidin deficiency	Severe	Rare
4A (Ferroportin disease)	Ferroportin 1	Dominant	Reticuloendothelial iron overload	Functional deficiency of ferroportin	Variable	Rare
4B (Ferroportin disease)	Ferroportin 1	Dominant	Parenchymal iron overload	Ferroportin shows defective binding of hepcidin	Variable	Rare

Ferritin is the major intracellular storage protein found in all cells, with the highest concentrations in the liver, spleen and bone marrow. Ferritin binds iron as a ferric complex within a protein shell. Each molecule can theoretically store up to 4500 atoms of ferric iron but, in practice, it is typically less than 2000 atoms. The protein shell is penetrated by channels through which ferrous iron enters to interact with a ferroxidase at the centre of the molecule [22]. Iron is able to exit after it has been reduced. This iron depot is readily accessible for hemoglobin synthesis. Serum ferritin concentrations are normally within the range 15–300 µg/L. They are lower in children than adults; from puberty to middle age, mean concentrations are higher in men than in women [23]. A close relationship exists between the total amount of storage iron and serum ferritin concentration in normal individuals [24], serum ferritin concentration of 1 µg/L being equivalent to approximately 8 mg stored iron.

## 8. Response to Iron Depletion

Increased needs for iron are met initially by increased release of iron from ferritin. Both heme and non-heme iron absorption are inversely related to serum ferritin concentrations which reflect iron reserves [25]: Absorption of dietary iron increases as ferritin depots decrease. Intestinal uptake and transfer responds to iron depletion in humans at serum ferritin concentrations of approximately 60 μg/L [26]. If absorption is not adequate, tissue iron stores are slowly depleted, which is reflected in a reduction in circulating transferrin saturation. As a result, the delivery of iron to functional sites decreases and iron-dependent functions, such as erythropoiesis, become impaired, leading to a decrease in hemoglobin concentration and the development of anemia.

## 9. Inborn Errors of Iron Metabolism

A number of sequence variations affect the genes coding for proteins involved in iron metabolism. The majority of these genetic changes need to be present in two corresponding chromosomes (autosomal recessive). Although heterozygotes (*i.e.*, individuals with one normal and one aberrant gene) have altered iron metabolism, this does not appear to affect their iron requirements or predispose them to excessively accumulate iron. Hereditary or Genetic Hemochromatosis is one of the most common single gene disorders found in populations of North European origin. It is an autosomal recessive disease caused mainly by mutation of the gene coding for the HFE protein [27]. It results in excessive absorption of dietary iron, causing high levels of iron to accumulate in the body. This can cause organ damage, leading to clinical manifestations including diabetes, arthritis, and cirrhosis of the liver [28]. Two common variants of this gene, C282Y and H63D, have been identified. In the U.K., over 90% of patients with hereditary hemochromatosis are homozygous for C282Y. In Europe, the highest allele frequency of C282Y (10%) is found in Ireland, followed by the U.K., Brittany, and Scandinavia (around 8%); and the lowest in Italy (0.5%) [29,30]. The variant is virtually absent in populations of non-European origin. The clinical penetrance of homozygosity for C282Y is highly variable and the majority of people with this genotype never become ill as a result of iron overload [31,32,33]. The H63D variant is more widespread worldwide and has a less defined role in predisposing towards iron loading. Most compound heterozygotes do not develop iron overload [34]. Other types of genetic hemochromatosis are outlined in Table 2.

“African Iron Overload” is caused by an unidentified genetic defect in iron metabolism combined with increased exposure to iron from contamination of drinks (e.g., beer) or food prepared or stored in ungalvanized steel containers or iron cooking pots. In contrast to the hemochromatoses, both heterozygotes and homozygotes appear to be affected [35]. 

## 10. The Effect of Infection and Inflammation on Iron Metabolism

Acute and chronic inflammation affects the systemic distribution and turnover of iron: deposition of iron in tissue ferritin is increased, and availability of iron for distribution to functional sites, as well as gastrointestinal iron absorption, is reduced; concentrations of circulating iron are decreased and those of ferritin increased. This paradoxical situation, of red cell and systemic functional iron deficiency accompanied by increased systemic and macrophage iron deposits, can become sustained with chronic inflammatory conditions and is known as the anemia of chronic disease. Infection and inflammation are accompanied by an acute phase response which involves the hepatic synthesis and release of a series of proteins known as acute phase reactants. These include hepcidin, the key regulator of iron absorption and of its release from macrophages and hepatocytes [36], contributing to the development of anemia of inflammation by reducing iron absorption and preventing the release of iron from macrophages. Ferritin also is an acute phase reactant. In the anemia of chronic disease, serum ferritin concentrations are higher than those of individuals with similar levels of tissue iron deposits but without infection and inflammation. Chronic disease is likely to be a significant confounder in population studies, and transient disturbances of iron metabolism in response to intercurrent infections need to be considered when interpreting the standard markers of iron metabolism. In developing countries, poverty, malnutrition, and infection are associated with the acute phase response and a correspondingly high prevalence of anemia of chronicdisease.

## 11. Requirements

Dietary reference values (DRVs) for iron are derived from limited data [37,38,39,40]. They are based on estimates of the amount of iron required to replace basal and menstrual iron losses, and for growth. The U.K. estimates are based on an assumed absorption of 15% from the diet. This percentage is derived from short-term studies carried out in iron replete individuals in whom iron absorption would be down regulated. Such studies do not allow for adaptive responses that occur over a longer time period than that needed for a single meal study, nor for the nature of this adaptation in response to systemic needs for iron. The DRVs for iron may be too high (particularly for girls and women of reproductive age) because the assumptions about the amount of iron absorbed and the degree of intestinal adaptation are cautious. There are currently insufficient *new* data to inform a reassessment of the DRVs for iron, however, improved understanding of iron metabolism could enable a reappraisal of existing data (Table 3 and Table 4).

**Table 3:** Dietary reference values for iron mg/day (µmol/day ^1^) [37].

AGE	Lower reference nutrient intake (LRNI)	Estimated average requirement (EAR)	Reference nutrient intake (RNI)
0–3 months	0.9 (15)	1.3 (20)	1.7 (30)
4–6 months	2.3 (40)	3.3 (60)	4.3 (80)
7–9 months	4.2 (75)	6.0 (110)	7.8 (140)
10–12 months	4.2 (75)	6.0 (110)	7.8 (140)
1–3 years	3.7 (65)	5.3 (95)	6.9 (120)
4–6 years	3.3 (60)	4.7 (80)	6.1 (110)
7–10 years	4.7 (80)	6.7 (120)	8.7 (160)
11–14 years (males)	6.1 (110)	8.7 (160)	11.3 (200)
11–14 years (females)	8.0 (140) ^2^	11.4 (200) ^2^	14.8 (260) ^2^
15–18 years (males)	6.1 (110)	8.7 (160)	11.3 (200)
15–18 years(females)	8.0 (140) ^2^	11.4 (200) ^2^	14.8 (260) ^2^
19–50 years (males)	4.7 (80)	6.7 (120)	8.7 (160)
19–50 years (females)	8.0 (140) ^2^	11.4 (200) ^2^	14.8 (260) ^2^
50+ years	4.7 (80)	6.7 (120)	8.7 (160)

^1^ 1 µmol = 55.9 µg; ^2^ COMA considered the distribution of iron requirements in women of child-bearing age to be skewed and the DRVs exclude those with high menstrual losses resulting in iron requirement above the EAR which is set at the 75th centile.

The DRVs for infants aged zero to six months may be redundant because infants are born with sufficient systemic iron to meet their functional needs for their first six months. Therefore, they are not dependent on breast milk or breast milk substitutes. Evidence from randomized controlled trials suggests that a delay in clamping the umbilical cord after birth, until it has stopped pulsing (about 2–3 min), is associated with higher systemic iron depots in the first six months of life. However it may also increase the risk of jaundice requiring phototherapy and it is not known if the beneficial effects are sustained [41].

**Table 4:** International dietary reference values for iron (mg/day).

UK [37]		USA and Canada [38]		FAO/WHO [40]			EU [39]	
**Age**	Recommended Nutrient Intake (based on 15% absorption)	**Age**	Recommended Dietary Allowance (based on 18% absorption)	**Age**	Recommended Nutrient Intake (based on 15% absorption)	Recommended Nutrient Intake (based on 10% absorption	**Age**	Population Reference Intake (based on 15% absorption)
0–3 m	1.7	-	-	-	-	-	-	-
4–6 m	4.3	0–6 m ^1^	0.27	-	-	-	-	-
7–9 m	7.8	-	-	-	-	-	-	-
10–12 m	7.8	7–12 m ^2^	11.0	6–12 m ^5^	6.2	9.3	6–12 m ^5^	6.2
1–3 y	6.9	1–3 y	7.0	1–3 y	3.9	5.8	1–3 y	3.9
4–6 y	6.1	4–8 y	10.0	4–6 y	4.2	6.3	4–6 y	4.2
7–10 y	8.7	-	-	7–10 y	5.9	8.9	7–10 y	5.9
MALES								
11–14 y	11.3	9–13 y	8.0	11–14 y	9.7	14.6	11–14 y	9.7
15–18 y	11.3	14–18 y	11.0	15–17 y	12.5	18.8	15–17 y	12.5
19–50 y	8.7	19–50 y	8.0	18+ y	9.1	13.7	18+ y	9.1
50+ y	8.7	50+ y	8.0	-	-	-	-	-
FEMALES								
11–14 y	14.8	9–13 y ^3^	8.0	11–14 y ^6^	9.3	14.0	11–14 y ^6^	9.3
15–18 y	14.8	14–18 y ^3^	15.0	11–14 y	21.8	32.7	11–14 y	21.8
19–50 y	14.8	19–50 y	18.0	15–17 y	20.7	31.0	15–17 y	20.7
50+ y	8.7	50+ y	8.0	18+ y	19.6	29.4	18+ y	19.6
-	-	Pregnancy ^4^	27.0	postmenopausal	7.5	11.3	postmenopausal	7.5
-	-	Lactation (14–18 y)	10.0	lactating	10.0	15.0	lactating	10.0
-	-	Lactation (19–50 y)	9.0	-	-	-	-	-

m: months; y: years; ^1^ No functional criteria of iron status have been demonstrated that reflect response to dietary intake in young infants. Thus, recommended intakes of iron are based on an Adequate Intake (AI) that reflects the observed mean iron intake of infants principally fed human milk; ^2^ Based on 10% absorption; ^3^ Based on assumption that girls younger than 14 years do not menstruate and that all girls 14 years and older do menstruate. For girls under age 14 who have started to menstruate, it would be appropriate to consider a median menstrual loss of 0.45 mg/day of iron. Therefore, the requirement is increased by approximately 2.5 mg/day of iron; ^4^ The bioavailability in the first trimester is as estimated for non-pregnant females, in the second and third trimesters, it is increased to 25%; ^5^ Bioavailability during this period varies greatly; ^6^ Non-menstruating.

## 12. Iron Status

Iron status describes whether an individual has too little, enough or too much, iron in their body for their needs. A number of hematological and biochemical markers are used to assess iron deficiency, adequacy, or excess (Table 5). The markers are categorized according to whether they represent a functional use of iron (hemoglobin), a role in the synthesis of hemoglobin (zinc protoporphyrin), supply of iron to tissues (iron bound to transferrin), iron depots in tissues (serum ferritin) or tissue needs for iron (serum transferrin receptors) (Table 6). No single marker of iron metabolism is considered ideal for the assessment of iron deficiency or excess as all the individual indices have limitations in terms of their sensitivity and specificity. However, in the SACN report [1], and in agreement with international practice [42], hemoglobin (functional iron) and serum ferritin (iron depots) were considered to be the most useful indicators of iron deficiency, adequacy, and excess. The WHO [43] criteria for identification of anemia, irrespective of cause, are; hemoglobin concentrations of: 110 g/L in children under 5 years; 115 g/L in children 5–11.99 years; 120 g/L in children 12–14.99 years and non-pregnant females over 15 years; 130 g/L in males over 15 years. The WHO criteria used to define depleted storage iron are serum ferritin concentrations of: <12 µg/L in children under 5 years; <15 µg/L in males and females over 5 years.

**Table 5:** A conceptual spectrum of iron status.

**Iron excess**	Cellular and tissue architectural and functional damage
	Increased tissue hemosiderin from degradation of ferritin
	Increased ferritin depots
	Reduced expression of transferrin receptors
**Iron adequacy**	Reduced intestinal uptake and transfer of iron
	Increased hepcidin
**Iron deficiency**	Reduced hepcidin
	Increased expression of transferrin receptors
	Mobilization of depots, reduced ferritin levels
	Increased intestinal uptake and transfer of iron (possibly induced at serum ferritin levels <60 µg/L)
	Reduced saturation of serum transferrin
	Functional defects in iron dependent activities
	Defective hemoglobin synthesis (increased zinc protoporphyrin)
	Reduced hemoglobin (anemia)
	Impaired muscle metabolism
	Secondary functional defects in the metabolism of other nutrients
	Cellular and tissue architectural and functional damage

The reference ranges for markers of iron metabolism define iron sufficiency. They do not define iron deficiency or iron excess as the thresholds selected for use are not based on functional defects. For example, low serum ferritin concentrations indicate low iron depots in tissues but they do not necessarily represent a functional deficiency of iron. It is not clear at which level, above or below the reference range for serum ferritin, there is an increased risk of an adverse outcome. Similarly, the thresholds used to define anemia do not correspond to concentrations of hemoglobin below which functional consequences of anemia occur. Individuals with values either above or below the reference ranges may still be healthy. The reference limits only indicate the possibility of iron depletion, deficiency, or excess. 

**Table 6:** Markers used for assessment of body iron status (adapted from BNF [44]).

Measurement	Representative reference range (adults)	Confounding factors	Diagnostic use
***Functional iron***			
Hemoglobin concentration		Other causes for anemia besides iron deficiency; a reciprocal relationship with iron stores should be expected in all anemias except in IDA.	Assess severity of IDA; response to a therapeutic trial of iron confirms IDA. Not applicable to assessment of iron overload
Males	130–180 g/L		
Females	120–160 g/L		
Red cell indices			
MCV *	84–99 fl	May be reduced in other disorders of hemoglobin synthesis (e.g., thalassaemia, sideroblastic anemias) in addition to ID.	
MCH	27–32 pg		
***Tissue iron supply***			
Serum iron	10–30 μmol/L	Normal short-term fluctuations mean that a single value may not reflect iron supply over a longer period. Both measures reduced in chronic disease.	Raised saturation of transferrin used to assess risk of tissue iron loading (e.g., in hemochromatosis or iron-loading anemias).
Saturation of transferrin	16–50%		
Serum transferrin receptor	2.8–8.5 mg/L **	Directly related to extent of erythroid activity as well as being inversely related to iron supply to cells.	Decreased saturation of transferrin, reduced red cell ferritin, increased zinc protoporphyrin, and increased serum transferrin receptors indicate impaired iron supply to the erythroid marrow.
Red cell zinc protoporphyrin *	<70 μmol/mol Hb (<80 mg/dL red cells)	Stable measures: reduced iron supply at time of red cell formation leads to increases in free protoporphyrin and hypochromic red cells, and reduced red cell ferritin. However, values may not reflect current iron supply	
Red cell ferritin (basic)	3–40 ag/cell		Serum transferrin receptors may have particular value in identifying early iron deficiency and, in conjunction with serum ferritin, distinguishing this from anemia of chronic disorders
% hypochromic red cells	<6%	May be increased by other causes of impaired iron incorporation into heme (e.g., lead poisoning, aluminium toxicity in chronic renal failure, sideroblastic anemias)	
***Iron in tissues***			
Serum ferritin			
Males	15–300 μg/L	Increased: as an acute phase protein and by release of tissue ferritins after organ damage.	All measures are positively correlated with iron stores except TIBC which is negatively correlated. Serum ferritin is of value throughout the range of iron stores. Quantitative phlebotomy, liver iron concentration, chelatable iron and MRI are of value only in iron overload. Bone marrow iron may be graded as absent, normal or increased and is most commonly used to differentiate ACD from IDA.
Females	15–200 μg/L		
Tissue biopsy iron-Liver (chemical assay)	3–33 μmol/g dry wt	Potential for sampling error on needle biopsy, especially when this is <0.5 mg, or liver is nodular. But remains the “gold standard” in iron overload.	
		
Bone marrow (Perls’ stain)			
		
Quantitative phlebotomy	<2 g iron		
		
Serum TIBC (may be measured directly or calculated from transferrin concentration)	50–70 μmol/L *		In IDA, a raised TIBC is characteristic.
Urine chelatable iron (after 0.5 g IM desferrioxamine)	<2 mg/24 h		
			
Non-invasive imaging	-		
MRI		Not yet sufficiently sensitive and reproducible for quantitation of normal levels of storage iron. Useful for detecting iron overload.	
SQUID (Magnetic susceptibility)		Sensitive, accurate and reproducible but only a few machines in the world.	

ACD: Anemia of chronic disease; Hb: hemoglobin; IDA: iron deficiency anemia; MCV: Mean corpuscular volume; MCH: Mean corpuscular hemoglobin; MRI: Magnetic resonance imaging; TIBC: Total iron binding capacity; * No internationally accepted cut-off values for MCV, TIBC, or ZPP have been developed because of analytical differences between laboratories and because these indicators can be influenced by variations in the conditions under which the blood samples were collected (e.g., fasting/non-fasting, time of day) and by the methods used for transportation, storage and processing; ** There is a major problem with the different units and reference ranges for the various assays in use [45,46].

## 13. Iron in the Diet

Iron is present in foods as heme or non-heme iron. Heme iron is found almost exclusively in foods of animal origin as hemoglobin and myoglobin. Non-heme iron is found in animal and plant tissues, fortified foods, and supplements. The most important determinant of dietary iron absorption is systemic iron need: more iron is absorbed from the diet in a state of iron deficiency and less is absorbed when iron depots are replete.

### 13.1. Bioavailability

Iron bioavailability refers to the proportion of iron that is taken up and transferred into the body by the intestinal mucosa and is used systemically. It is affected by the chemical form of iron. Heme iron is absorbed more efficiently from the diet than non-heme iron [47,48]. A number of dietary components have been shown to increase or reduce non-heme iron absorption from single test meals. The main enhancers of non-heme iron absorption are meat, and ascorbic acid found in fruit and vegetables. The main inhibitors of non-heme iron absorption are calcium, phytates in cereals and legumes, and phenolic compounds found in tea, coffee, and other beverages. However, single meal absorption studies do not take account of adaptive absorptive responses to qualitative and quantitative changes in the diet. Studies over longer periods indicate that single meal studies overestimate the effects of enhancers and inhibitors of iron absorption [49,50,51]. This might be due to interactions between the various ligands for iron and their combined influence on mucosal uptake, and to mucosal adaptation.

### 13.2. Models of Bioavailability

Several models have been developed to estimate iron bioavailability from different meals and diets based on the serum ferritin concentration of the individual, the type of iron (heme or non-heme), and that take into account the presence of enhancers or inhibitors of iron absorption [52,53,54]. They have a number of limitations as they are based on iron absorption from single meals which may overestimate the effects of enhancers and inhibitors and do not take account of dietary complexity and variability or long-term adaptation to iron absorption. For example, Beard *et al.* [55] compared a number of prediction equations to the change in serum ferritin concentration of women taking part in a feeding trial in the Philippines to assess the efficacy of iron fortified rice. There were highly significant differences in the predicted efficiency of iron absorption from six equations and none agreed with dietary iron utilization based on improvement in serum ferritin concentration.

### 13.3. Epidemiology of Dietary Modulators and Iron Status

The effects of dietary modulators of non-heme iron absorption on markers of iron status (usually hemoglobin and serum ferritin concentration) are difficult to ascertain in epidemiological studies due to the difficulty of obtaining accurate exposure data because of the quality of dietary assessments, limited food composition data for some modifiers of iron absorption, and interactions between enhancers and inhibitors of non-heme iron absorption. Observational studies are also affected by a number of confounding factors such as disease which can raise serum ferritin concentrations. Such studies have not shown a clear relationship between intakes of total iron or enhancers and inhibitors of iron absorption and systemic markers of iron status, although most cross-sectional studies have reported better iron status with increased meat intake [56,57,58,59] and heme iron intake [60,61,62].

### 13.4. Prospective and Intervention Studies

Evidence from a limited number of prospective studies [e.g., 63,64] suggest that dietary inhibitors and enhancers of iron absorption do not substantially influence iron status. Long term intervention studies have also, overall, not shown a corresponding change in markers of iron status [e.g., 65,66,67]. A measurable effect of dietary modulators may only be observed in individuals with increased systemic iron needs and, as a consequence, higher absorptive capacity. Most intervention studies were carried out in iron replete western populations who are less likely to have a physiological response to additional dietary iron. It is also possible that the lack of effect on serum ferritin concentration is because of relative insensitivity of serum ferritin concentration to changes in iron depots. This raises uncertainties regarding the importance of dietary advice in the U.K. and similar countries to maximize iron absorption: for example, eating cereal sources of iron with foods rich in vitamin C or avoiding drinking tea with meals.

### 13.5. Anemia and Iron Deficiency

There are no data to indicate that the bioavailability of dietary iron is a significant factor in the pathogenesis of anemia and iron deficiency in the U.K. population. U.K. diets contain a broad range of foods containing iron and various enhancers and inhibitors of iron absorption. Consequently, the bioavailability of dietary iron may have little influence on iron status in the U.K. population. Iron bioavailability may become a limiting factor in certain circumstances, for example, for individuals with an increased need of iron, particularly when the iron content of the diet is low [63]. The effects of enhancers and inhibitors of iron absorption may also be more important in developing countries where populations are at greater risk of iron intakes insufficient to meet requirements since diets are plant based, more limited and monotonous, contain higher levels of inhibitors, lower levels of enhancers, and less heme iron. Under these circumstances, the imbalance between requirements and absorption may lead to iron deficiency.

### 13.6. Fortification

Fortification of foods with iron has been the main approach used to increase the iron supply of the U.K. population. Iron fortification of white and brown wheat flour, to replace iron lost during processing, is mandatory in the U.K. A number of other foods, including breakfast cereals and breast milk substitutes are fortified on a voluntary basis. The iron compounds used for fortification of foods vary in their availability for intestinal uptake. Iron compounds which are relatively soluble, such as ferrous sulfate, are not widely used for food fortification because they can cause unfavorable organoleptic changes during prolonged storage. Although elemental iron powders are less soluble they are more commonly used for fortification of foods, especially cereal products. This is because they are relatively inert and, since they do not react with the food vehicle used for fortification, they have a longer shelf life; they are also less expensive than other iron fortification compounds. Although iron fortified foods, especially cereals, make a substantial contribution to iron intakes in the U.K., evidence from efficacy trials and from countries with national fortification policies, e.g., Denmark [59], Venezuela [68] and Brazil [69], suggests that foods fortified with elemental iron make little practical contribution to improving iron status, even in individuals with increased systemic iron needs, which is probably due to their low solubility and consequently low intestinal uptake. The usefulness of iron fortified breast milk substitutes in improving iron status of infants is also uncertain.

### 13.7. Supplements

Many non-heme iron supplements are available over the counter from chemists, supermarkets and health food shops. The most common forms are ferrous sulfate, ferrous fumarate, ferrous gluconate, ferrous glycine sulfate and iron polysaccharide [70]. The bioavailability differs but all are generally better absorbed than slow-release capsules or multivitamin/multimineral supplements [71]. Iron supplements are usually used as a short-term measure to provide extra iron when iron levels are low. Commercially available prophylactic doses used to prevent deficiency usually range between 7–50 mg/day. Supplemental intakes above the Guidance Level (an approximate indication of the amount of a nutrient that would not be expected to cause any adverse effects) of 17 mg/day are not advised in the U.K. (Expert Group on Vitamins and Minerals.) [72]. During pregnancy, iron supplements are recommended for women with hemoglobin concentrations outside the normal U.K. range for pregnancy (*i.e.*, 110 g/L during the first trimester and 105 g/L at 28 weeks) [73].

## 14. Consequences of Iron Deficiency

Causes of iron deficiency include inadequate intakes of iron, impaired absorption, and increased blood losses due to menstruation or gastrointestinal disease [74,75,76]. Increased systemic need for iron leads to mobilization of iron depots from macrophages or hepatocytes and upregulation of iron absorption. Progressive iron deficiency leads to anemia and reduced numbers of circulating precursor red cells and iron-dependent functions are affected. Anemia has been reported to have adverse effects on physical work capacity, pregnancy outcomes, and cognitive, motor and behavioral development in children.

### 14.1. Work Capacity

Evidence from animal and human studies suggests that decreases in hemoglobin concentration are associated with impairments in various aspects of physical work capacity (aerobic capacity, endurance capacity, energetic efficiency, voluntary activity, work productivity) [77]. The available data suggest functional defects associated with physical work capacity at hemoglobin concentrations at or below 110–120 g/L and ferritin concentrations at or below 16–20 µg/L. Human studies suggest that aerobic capacity is reduced at hemoglobin concentrations below about 110 g/L; however there is no clear evidence that iron deficiency in the absence of anemia has adverse effects on aerobic capacity [e.g., 78,79]. There is a limited amount of evidence suggesting that iron deficiency in the absence of anemia (hemoglobin >120 g/L; serum ferritin <16 µg/L) might impair endurance capacity, but this needs further substantiation. Overall there are insufficient data to assess the effects of iron deficiency or iron deficiency anemia on energetic efficiency, voluntary activity, or work productivity. There are a number of limitations with many of the human studies which assessed the relationship between iron and physical work capacity, including poor characterization of iron deficiency and an assumption that anemia is caused by iron deficiency. Additionally, most studies were carried out in developing countries where there are multiple nutritional and socioeconomic deprivations which could confound the relationship between iron and physical work capacity. Clear thresholds associated with adverse outcomes cannot be determined because the data are presented discontinuously.

### 14.2. Maternal Iron Status and Pregnancy Outcome

Data from observational studies have suggested that maternal hemoglobin concentrations at either the low or high end of the distribution during pregnancy are associated with increased risk of adverse birth outcomes including low birth weight, preterm birth, and perinatal mortality [80]. However these are not necessarily causally related to iron supply or nutrition. The physiological changes that occur during pregnancy, such as plasma volume expansion and hemodilution, make it difficult to interpret the markers of iron metabolism during this time [81]. High hemoglobin concentrations during pregnancy are generally not caused by high intakes of dietary or supplemental iron but are the result of inadequate plasma volume expansion, which is also associated with adverse birth outcomes [82,83,84]. Intervention studies of routine iron supplementation during pregnancy have not reported beneficial or adverse effects on pregnancy outcomes [85]. Evidence supports the recommendation made by the National Institute for Clinical Excellence (NICE) [73], that iron supplementation should not be offered routinely to all pregnant women but should be considered for women identified with hemoglobin concentrations below 110 g/L in the first trimester and 105 g/L at 28 weeks.

### 14.3. Cognitive, Motor and Behavioral Development in Children

Observational studies show that iron deficiency and iron deficiency anemia are usually associated with many psychosocial, economic and biomedical disadvantages, which can independently affect development. Iron deficient anemic young children usually have poorer development concurrently and in the future than non-anemic children. Measured and unmeasured environmental variables could possibly explain these findings.

Evidence from randomized controlled trials of iron supplementation suggests that iron deficiency anemia is a cause of poor motor development in children in the first three years of life but the long term effects are unknown [86,87]. There is insufficient evidence from rigorous randomized controlled trials to determine whether iron deficiency or iron deficiency anemia affects cognitive or language development in children three years or under. The relatively short duration of follow up in the trials may explain the lack of observed effect. There is evidence to suggest that iron treatment has beneficial effects on cognitive development in anemic older children, however it is not known whether these benefits are sustained [88,89,90]. Based on current evidence, it is not possible to derive thresholds of iron status at which cognitive, motor and behavioral development might be at risk, however risks appear to be lower at hemoglobin concentrations above 110 g/L.

There are a number of difficulties in interpreting and comparing the data examining adverse effects of iron deficiency and iron deficiency anemia. This is because most studies have been conducted in developing countries where populations are associated with multiple nutritional deficiencies combined with social and economic deprivations. All these factors, which usually accompany iron deficiency, are potential confounders because they may be independently associated with adverse effects on physical work capacity, pregnancy outcomes, and cognitive, motor and behavioral development in children. Another difficulty is that iron deficiency and iron deficiency anemia is often poorly characterized in these studies: many only measure hemoglobin and assume this represents iron deficiency; another assumption is that dietary iron deficiency is the cause of this anemia rather than loss of iron secondary, for example, to blood loss. Additionally, sample sizes are small and different reference ranges and cut-off points have been used.

## 15. Consequences of High Iron Intake and Burden

Acute high doses of iron can cause intestinal mucosal damage and systemic toxicity [91]. Lower exposures may interfere with the intestinal uptake, transfer, and systemic use of copper and zinc [92,93]. High systemic iron burden is also associated with adverse effects arising from degradation of tissue ferritin and subsequent free radical damage of surrounding tissues [94]. In the U.K., the evidence for adverse effects of iron was considered insufficient to establish a safe upper level (SUL—an intake that can be consumed daily over a lifetime without significant risk to health; established when supported by adequate data). Instead, a guidance level (GL—GLs are less secure than SULs because they are derived from limited data) of 17 mg/day of supplemental iron (*i.e.*, in addition to dietary intake), based on gastrointestinal effects, was recommended for adults [72]. In the U.S., a tolerable upper intake level (UL—the Tolerable Upper Intake Level represents the highest level of daily nutrient intake that is likely to pose no risk of adverse health effects for almost all individuals in the general population) for total iron intake (from all sources) of 45 mg/day was set for adults, which was also based on gastrointestinal effects. A UL for iron has not been set in Europe as adverse gastrointestinal effects were not considered a suitable basis to establish a UL for iron from all sources and there were insufficient data regarding other risks.

It has been proposed that high iron intakes or high body iron burden may increase the risk of colorectal cancer [95], cardiovascular disease (CVD) [96,97], infection [97], neurodegenerative disorders [98], and inflammatory conditions [99]. The SACN report focused on the link between iron intakes/systemic iron, with colorectal cancer and CVD as these were considered to be the main issues of public health concern in the U.K. The report also considered effects of high exposures to iron on growth in iron replete children. Other conditions that have been associated with high iron intake/systemic iron, e.g., Alzheimer’s disease, Parkinson’s disease, arthritis, and diabetes mellitus, were only briefly considered.

### 15.1. Colorectal Cancer

There is a limited amount of epidemiological data on the association between iron intakes and high iron depots on colorectal cancer risk [100]. The available data suggest that: increased dietary intakes of total or heme iron might be associated with increased colorectal cancer risk, however confounding by other dietary and lifestyle factors is possible; high iron depots are not associated with increased colorectal cancer risk; and heterozygosity for hereditary hemochromatosis might be associated with increased colorectal cancer risk but it is not clear if this is related to iron. Overall, there are insufficient data on the association between colorectal cancer risk and dietary intakes of total iron, heme iron, iron status, or heterozygosity for hereditary hemochromatosis, to reach clear conclusions.

Meat, particularly red meat, is almost exclusively the source of heme iron. In their report, *Nutritional Aspects of the Development of Cancer* [2], COMA concluded that there was moderately consistent evidence, from cohort studies, of a relationship between red and processed meat consumption and colorectal cancer. The substantial body of prospective epidemiological data that has accumulated since the COMA report in 1998 consistently indicates an increased colorectal cancer risk associated with high intakes of red and processed meat [100]. Overall, the available epidemiological evidence suggests that red and processed meat intake is probably associated with increased colorectal cancer risk. The evidence for an increased colorectal cancer risk is not unequivocal since it is based on prospective observational studies, so effects of confounding by other dietary or lifestyle factors associated with meat consumption and colorectal cancer risk cannot be excluded. Although a number of plausible biological mechanisms have been proposed to explain the association between red meat and colorectal cancer risk, none are supported by robust evidence. It is not possible to identify if there is a dose-response or a threshold level of red and processed meat which may be associated with increased colorectal cancer risk because of a number of limitations in the data.

### 15.2. Cardiovascular Disease

The available epidemiological evidence on total iron intake or body iron and CVD does not suggest an association [101,102]. The evidence examining the association between heme iron intake and CVD risk is limited to a few studies which suggest overall that high intakes of heme iron are associated with increased CVD risk. However it is possible that this could be due to other components of meat (the main source of heme iron) associated with CVD risk, such as saturated fats or other dietary and lifestyle factors associated with meat intake. Studies of HFE heterozygosity and CVD risk suggest that C282Y heterozygotes (but not H63D heterozygotes) may be at increased risk of CVD, but there are insufficient data to reach clear conclusions [103].

### 15.3. Growth

Iron supplementation may have a negative effect on the physical growth of iron replete (hemoglobin >110 g/L; serum ferritin >12 µg/L) infants and children [87,104,105] but further studies are required to characterize this effect. 

### 15.4. Other Consequences

There is insufficient evidence to suggest that high iron intake or high iron depots increase the risk of diabetes mellitus in the general population or that homozygosity or heterozygosity for hereditary hemochromatosis increases diabetes risk. The limited amount of evidence for an association between iron intake and rheumatoid arthritis is inconclusive. There is no evidence that dietary iron is associated with Parkinson’s disease or Alzheimer’s disease.

## 16. Effect of Iron Deficiency and Excess on Immunity and Infection

Evidence from animal studies suggests that iron plays a role in immunity and infection. Human studies have shown that iron deficiency anemia (typically defined as hemoglobin <100 g/L plus 1 or more measure of iron deficiency) [106,107,108] and iron overload (due to multiple blood transfusions) impair some aspects of immune function [109,110]. However it is not known if these impairments increase susceptibility to infectious pathogens. 

### Supplements

Although it has been proposed that iron supplementation may decrease resistance to infection, evidence suggests that it does not increase the risk of non-diarrheal or respiratory tract infections in children but may increase diarrhea risk [104,111]. It is not clear if iron supplementation increases risk of malaria or risk of infectious diseases in areas where malaria incidence is high [112,113,114,115]. There is currently insufficient evidence to draw conclusions on the relationship between iron supplementation and HIV or tuberculosis. Most human studies on iron and infection have been conducted in developing countries where multiple nutrient deficiencies co-exist and which may also affect resistance to infection. There is no evidence to suggest that iron supplementation would have any effect on infectious disease incidence or morbidity in the U.K. However iron supplementation may have adverse effects in some subgroups of the population, for example, those with HIV or children at risk of diarrhea [115,116].

## 17. Dietary Iron Intakes and Status in the U.K.

The *National Diet and Nutrition Survey* (NDNS), in a series of separate cross-sectional surveys of different age groups, and the *Low Income Diet and Nutrition Survey* (LIDNS) [117,118,119,120,121,122,123,124] provide nationally representative data on iron intakes and iron status in the U.K. for the general population and low income populations respectively. However there are difficulties associated with using dietary surveys to assess the adequacy of nutrient intakes against DRVs, including reliability of food composition tables and misreporting of food consumption. Additionally, assessment of the adequacy of iron intakes against DRVs only takes limited account of the amount of iron absorbed from the diet. The WHO thresholds for iron deficiency (based on serum ferritin concentration) and anemia (based on hemoglobin concentration) were used to identify the prevalence of iron deficiency and iron deficiency anemia in the U.K. However, data on hemoglobin and serum ferritin values should be interpreted with caution since neither marker, alone or in combination, necessarily diagnose iron deficiency but indicate individuals at risk of deficiency.

Although the NDNS is now a continuous rolling programme of people aged 18 months and over living in the U.K. and intake data from year 1 (2008/9) have been published [125], they were not used to assess adequacy of intakes/status because the sample size of intake data is currently too small for robust subgroup analysis and the blood status data have not yet been published because the sample size is currently too small for meaningful analysis.

Data from the NDNS series and LIDNS show that iron fortified cereals, including bread, are the main contributors and contribute about half of the iron intake of most of the population in the U.K. (Table 7 and Table 8). The contribution that fortified foods make to the supply of absorbed iron available for uptake, transfer and systemic utilization, and their effect on iron status in the U.K. is not known. Meat and meat products and vegetables also make substantial contributions to dietary iron intakes. Although heme iron is more bioavailable than non-heme iron, the NDNS did not find any significant associations between heme iron and markers of iron status (serum ferritin and hemoglobin).

**Table 7:** NDNS—Contribution (%) of food types to average daily intake of total iron.

	Children		Young people		Adults		Adults 65+		Adults 65+	
	1½–4½		4–18 years		19–64 years		Free-living		Institutions	
	Males	Females	Male	Females	Males	Females	Males	Females	Males	Females
**Cereals**	49	48	55	51	44	45	48	47	50	50
*white bread*	8	8	11	11	*10*	*8*	10	9	10	10
*wholemeal bread*	3	3	2	2	*3*	*3*	7	7	5	6
*soft grain and other bread*	-	-	-	-	*3*	*3*	-	-	-	-
*whole grain and high fibre breakfast cereals*	11	10	12	9	*12*	*13*	10	12	8	9
*other breakfast cereals*	10	10	17	14	*6*	*7*	5	5	9	8
*biscuits, buns, cakes, pastries*	5	5	6	8	*4*	*5*	8	8	10	10
**Milk and milk products**	6	6	3	3	1	1	3	4	4	5
**Eggs and egg dishes**	2	3	2	2	3	3	3	3	4	4
**Fat spreads**	0	0	0	0	0	0	0	0	0	0
**Meat and meat products**	14	14	14	13	19	**15**	**18**	16	17	15
**Fish and fish dishes**	2	2	1	2	2	3	3	2	2	2
**Vegetables (excluding potatoes)**	7	7	7	8	9	11	8	10	8	8
**Potatoes and savory snacks**	7	7	7	7	7	8	7	7	5	5
**Fruit and nuts**	3	3	1	2	2	3	3	3	3	3
**Sugars, preserves, confectionery**	4	3	4	4	2	2	1	1	1	1
**Drinks**	3	3	1	2	7	6	3	3	1	1
**Misc**	2	2	2	2	3	3	3	4	5	6
*Total no respondents (w)*	*-*	*-*	*-*	*-*	*833*	*891*	*540*	*735*	*93*	*319*
*Total no respondents (unw)*	*848*	*827*	*856*	*845*	*766*	*958*	*632*	*643*	*204*	*208*

* includes soft drinks, alcoholic drinks, tea, coffee and water; ** includes powdered beverages (except tea and coffee), soups, sauces, condiments and artificial sweeteners.

**Table 8:** The *Low Income Diet and Nutrition Survey* (LIDNS)—Contribution (%) of food types to average daily intake of total iron.

	Children (2–10 years)		Adults (19 years and over)	
	Boys	Girls	Men	Women
**Cereals and cereal products**	53	49	40	41
**Milk and milk products**	2	2	1	1
**Eggs and egg dishes**	2	2	4	3
**Fat spreads**	0	0	0	0
**Meat and meat products**	17	16	23	21
**Fish and fish dishes**	1	2	2	3
**Vegetables excluding potatoes**	7	8	10	10
**Potatoes and savory snacks**	10	11	8	9
**Fruit and nuts**	1	2	2	2
**Sugars, preserves and confectionery**	3	3	2	2
Drinks *	1	2	5	4
Miscellaneous **	2	2	3	4
*Total no. respondents (unw)*	*439*	*493*	*946*	*1850*

* includes soft drinks, alcoholic drinks, tea, coffee and water; ** includes powdered beverages (except tea and coffee), soups, sauces, condiments and artificial sweeteners.

### 17.1. Total Iron Intakes

Average iron intakes in the general population are near (>90%) or above the reference nutrient intake (RNI- the RNI represents the amount of a nutrient that is likely to meet the needs of 97.5% of the population) for most population groups in the U.K. (Table 9). Intakes below 90% of the RNI were reported in a high proportion of children aged 1½–3½ (73–81%), girls aged 11–18 years (60%) and women aged 19–49 years (66–87%). Population groups with substantial proportions below the lower reference nutrient intake (LRNI—the LRNI represents the amount of a nutrient that is likely to meet the needs of 2.5% of the population) were children aged 1½–3½ (12–24%), girls aged 11–18 years (44–48%) and women aged 19–49 years (25–40%).

**Table 9:** NDNS—Total mean (median) iron intake from all sources and food sources.

	MALE				FEMALE			
**Age** (years)	Mean (median) intake—all sources, mg/day	Mean (median) intake—food sources, mg/day	*Base (w)*	*Base (unw)*	Mean (median) intake from all sources, mg/day	Mean (median) intake food sources, mg/day	*Base (w)*	*Base (unw)*
**Children**	**5.7 **(5.4)	**5.5 **(5.4)			**5.4 **(5.0)	**5.2 **(5.0)		
**1.5–4.5**			*-*	*848*			*-*	*827*
1.5–2.5 *	5.0 (4.7)	4.9 (4.7)	*-*	288 **	5.0 (4.7)	4.9 (4.7)	*-*	288 **
2.5–3.5 *	5.6 (5.4)	5.4 (5.3)	*-*	303 **	5.6 (5.4)	5.4 (5.3)	*-*	303 **
3.5–4.3	6.2 (5.9)	6.1 (5.9)	*-*	*250*	5.9 (5.5)	5.6(5.5)	*-*	*243*
**Young people 4–18**	**10.5 **(9.9)	**10.4 **(9.8)	*3331*	*856*	**8.5 **(8.0)	**8.3 **(7.9)	*3159*	*845*
4–6	8.3 (8.0)	8.2 (7.9)	*1134*	*184*	7.4 (7.1)	7.3 (7.1)	*656*	*171*
7–10	9.8 (9.3)	9.7 (9.3)	*912*	*256*	8.5 (8.2)	8.4 (8.2)	*866*	*226*
11–14	10.8 (10.4)	10.8 (10.4)	*870*	*237*	9.1 (8.6)	8.8 (8.4)	*821*	*238*
15–18	12.6 (11.7)	12.5 (11.6)	*861*	*179*	8.9 (8.2)	8.7 (8.0)	*816*	*210*
**Adults 19–64**	**14 **(12.9)	**13.2 **(12.6)	*833*	*766*	**11.6 **(10.0)	**10.0 **(9.6)	*891*	*958*
19–24	11.5 (11.3)	11.4 (11.2)	*108*	*61*	10.0 (9.3)	8.8 (9.1)	*104*	*78*
25–34	13.9 (12.8)	13.0 (12.5)	*219*	*160*	9.8 (9.0)	9.2 (9.0)	*210*	*211*
35–49	14.1 (13.2)	13.7 (13.1)	*253*	*303*	12.9 (10.5)	10.2 (10.1)	*318*	*379*
50–64	15.2 (13.6)	13.6 (13.3)	*253*	*242*	12.3 (11.0)	10.9 (10.6)	*259*	*290*
**Adults 65 and over**								
*Free-living*	**11.6 **(10.6)	**11.0 **(10.5)	*540*	*632*	**8.9 **(8.4)	**8.6 **(8.3)	*735*	*643*
65–74	11.9 (10.6	11.1(10.5)	*353*	*271*	9.3 (8.7)	9.0 (8.6)	*409*	*256*
75–84	11.1 (10.7)	10.8 (10.5)	*160*	*265*	8.5 (8.1)	8.4 (8.1)	*249*	*217*
85+	10.6 (9.7)	10.4 (9.7)	*26*	*96*	7.9 (7.6)	7.7 (7.5)	*77*	*170*
*Institutionalized*	**9.6 **(9.3)	**9.6 **(9.3)	*93*	*204*	**8.3 **(7.9)	**8.2 **(7.9)	*319*	*208*
65–84	9.6 (9.2)	9.6 (9.2)	*57*	*128*	8.7 (8.1)	8.6 (8.1)	*144*	*91*
85+	9.7 (9.3)	9.6 (9.3)	*36*	*76*	8.0 (7.7)	7.8 (7.6)	*174*	*117*

* Data reported for boys and girls combined ** Half of the base figure for the sum of boys and girls as data combined in report.

In low income groups average daily intakes were above the RNI for all males. For females, average intakes of iron were at or above the RNI for girls aged 2–10 years and women aged 65 years and over; mean daily intakes were below the RNI for girls aged 11–18 years (63% of RNI) and women aged 19–49 years (about 60% of RNI). A high proportion of females aged 11–49 years (39% of girls aged 11–18 years; 50% women 19–49 years) had intakes below the LRNI.

### 17.2. Hemoglobin

Groups with the highest prevalence of hemoglobin concentrations below WHO thresholds for anemia in the general U.K. population were adults aged 65 years and above living in institutions (39–52%), free-living adults aged 75 years and over (13–38%), and girls aged 4–6 years (15% based on higher threshold of 115 g/L; 9% based on lower threshold of 110 g/L). In low income groups the highest proportions of adults with hemoglobin concentrations below the WHO cut-offs were men aged 65 years and above (20%).

### 17.3. Serum Ferritin

In the general population substantial proportions of children aged 1½–4½ years, girls aged 11–18 years, women aged 19–24 years and 35–49 years, and free living adults 75 years and over, had serum ferritin concentrations below WHO thresholds indicating an increased risk of iron deficiency. In low income groups, women aged 19–49 years were at greatest risk of iron deficiency.

### 17.4. Iron Deficiency Anemia

In the general population, risk of iron deficiency anemia (hemoglobin and serum ferritin concentration below WHO thresholds) was highest (5–6%) for children aged 1½–2½ years, girls aged 15–18 years, women aged 35–49 years, men 65 years and over living in institutions, and free living adults aged 85 years and over. In low income groups, a substantial proportion of women aged 19–39 years were at risk of iron deficiency anemia (9–11%).

Although data from the NDNS and LIDNS suggest that considerable proportions of some population groups may have iron intakes below amounts required to meet their requirements, this is not clearly consistent with the iron status data which suggests that for 95% of the general population, current intakes are adequate to maintain their iron status above internationally accepted criteria for iron deficiency anemia. The mismatch between the iron intake and iron status data suggests that the DRVs for iron may be too high. The DRVs are based on limited data and may not take full account of absorptive adaptation to increased iron needs.

The NDNS and LIDNS both broadly show that women aged 15–50 years are at increased risk of iron deficiency anemia which is consistent with iron losses in this age group due to menstrual blood loss. More women of reproductive age from low income groups are at risk of iron deficiency anemia compared to those in the general population. The reasons for this are not clear as mean iron intakes are similar. It is possible that intakes are insufficient to compensate for higher pregnancy burden and/or greater ill health in women from low income groups. Iron deficiency anemia observed in some adults aged 65 years and over might be caused by decreased absorption of dietary iron due to gastric atrophy or increased blood loss due to gastrointestinal disease or medication. There are no data to clarify the etiology of iron deficiency in the U.K. population. 

Although data from NDNS and LIDNS suggest that iron intake and iron status in the U.K. may be of public health concern for toddlers, women of reproductive age, and adults aged 65 years and over, this is dependent on the confidence placed on the DRVs for iron intake which are based on cautious assumptions and on iron status criteria for iron deficiency and iron deficiency anemia which are not based on functional defects.

## 18. Potential Impact of Reducing Total Red Meat Consumption on Iron and Zinc Intake

A modeling exercise (based on intake data from the 2000/1 NDNS of adults age 19–64 years) to explore the potential effect of reducing total red (including processed) meat consumption on intakes of iron and zinc, suggests that red meat makes a greater contribution to total zinc intake from all foods (32% for men; 27% for women) than to total iron intakes (12% for men; 9% for women). In 2000/1 the average consumption of total red meat (consumers of red meat only) in the U.K. was approximately 70 g/day (cooked weight) (88 g/day, men; 52 g/day, women). This is lower than the previous estimate of average total red meat intake of 90 g/day cited in the 1998 COMA report [2] because the COMA figure included non-meat components of composite dishes such as meat products (e.g., sausage rolls, pies) and meals containing red meat (e.g., lasagna, stew) resulting in an overestimation of red meat consumption. In 2000/1, the average total red meat intake of consumers in the 75th percentile of the distribution of intakes was approximately 94 g/day cooked weight (115 g/day for men; 70 g/day for women).

Preliminary findings from the first year (2008/9) of the NDNS rolling program suggest that current consumption of red meat is approximately 10 g/day higher than it was in 2000/1. However, these findings should be interpreted with caution because there are important insecurities in the data (see full SACN report for further details).

The modeling data suggest that reducing the red and processed meat intakes of consumers in the upper ranges of the distribution down to an average of 80 g/day would have a minimal impact on the proportion of individuals with average intakes below the LRNI for iron and zinc. Further reductions in the total red meat intake of consumers to an average of 70 g/day would have little effect on iron intakes but the proportion of men with intakes of zinc below the LRNI may increase from 3.7% to 5.5%.

## 19. Summary

There are a number of uncertainties which complicate a risk assessment of iron and health. The main sources of uncertainty are: lack of definitive data on the amounts of heme and non heme in the diet, difficulties in assessing dietary iron intakes, poor correlation between intakes and systemic iron load, difficulty in measuring adaptive and functional responses to variations in iron intake, uncertain and possibly conservatively high estimates of DRVs, lack of sensitive and specific markers to assess iron deficiency or excess, lack of consistent quality control and reference values in measurement of customary markers of iron status, inadequate characterization of the role of iron deficiency anemia and the relative role of iron deficiency and other causes of anemia in studies investigating the health consequences of iron deficiency, small sample sizes in most studies, and confounding by other dietary and lifestyle factors and by alterations in iron metabolism in response to infection. All these uncertainties make it difficult to determine dose-response relationships or confidently characterize the risks associated with iron deficiency or iron excess.

## 20. Recommendations

The following are the main recommendations of the SACN report on Iron and Health [1]:

- It is important to ensure that the UK population has a safe and adequate supply of iron to meet physiological requirements. It is recommended that a public health approach to achieving adequate iron status should emphasise the importance of a healthy balanced diet that includes a variety of foods containing iron. Such an approach is more important than focusing on particular inhibitors or enhancers of the bioavailability of iron from diets.
- While substantial proportions of the UK population appear to have iron intakes below dietary recommendations for iron, this is not clearly consistent with the low prevalence of poor iron status (see next paragraph). This might be because there are important uncertainties in the DRVs for iron intake which may be too high, particularly for girls and women of reproductive age. It is recommended that the DRVs for iron should be reviewed when more data become available (see research recommendations).
- Although there are many uncertainties in the data, about 95% of the UK population is iron replete (haemoglobin and serum ferritin concentrations above the WHO thresholds used to define iron deficiency and anaemia). However some population groups may be at risk of iron deficiency anaemia (WHO criteria for iron deficiency are serum ferritin concentrations below the following thresholds: children under 5 years, 12 µg/L; males and females 5 years and over, 15 µg/L. WHO criteria for anaemia are haemoglobin concentrations below the following thresholds: children under 5 years, 110 g/L; children 5–11.99 years, 115 g/L; children 12–14.99 years and non-pregnant females over 15 years, 120 g/L; males over 15 years, 130 g/L). These include toddlers, girls and women of reproductive age (particularly those from low income groups) and some adult groups aged over 65 years. It is recommended that health professionals be alert to the increased risk of iron deficiency anaemia in these groups. Those with signs and symptoms suggestive of iron deficiency anaemia should receive appropriate clinical assessment and advice, including dietary advice on how to increase their iron intakes and to consider use of iron supplements if required.
- Current evidence does not support routine iron supplementation of pregnant women but this should be kept under review. The recommendation by NICE (2008) is therefore supported, that iron supplementation should not be offered routinely to all pregnant women but should be considered for women identified with haemoglobin concentrations below 110 g/L in the first trimester and 105 g/L at 28 weeks.
- Red and processed meat is a source of iron in the diet of the UK population. COMA reported possible links between red and processed meat consumption and colorectal cancer risk in 1998 and the evidence that has accumulated since then generally supports this association. However, it is not possible to quantify the amount of red and processed meat that may be associated with increased colorectal cancer risk because of limitations and inconsistencies in the data. It may be advisable for adults with relatively high intakes of red and processed meat (e.g., it is estimated that those above the 75th percentile consume over 90 g/day) to consider reducing their intakes. Evidence from a theoretical modelling exercise indicates that a reduction in the red and processed meat intakes of high consumers, to the population average for adult consumers (about 70 g/day cooked weight in 2000/01), would have little impact on the proportion of the adult population with iron intakes below the LRNI. However, this estimate is based on data from 2000/01 and will need to be kept under review.

*Research* *Recommendations*

The following are the main recommendations of the SACN report [1] for further research:

- A more coordinated approach to research on iron in the U.K. and elsewhere is required to characterize iron status involving harmonization of reference ranges and analytical quality control for markers of iron metabolism. Consistent study designs and protocols will enable better characterization of function thresholds in relation to iron sufficiency, deficiency, or excess. This would improve the cost benefits of the research and enable research findings to be more relevant to public health needs.
- Good quality dose-response data are required to enable a reassessment of the dietary reference values for iron. Knowledge of the systemic regulation and mediation of iron homeostasis should be applied to characterize better the responses to increased and reduced systemic needs for iron and the development, or better validation, of existing markers used to assess the adequacy of iron status in populations and individuals.
- Future studies assessing the relationship between iron excess and chronic disease should employ a standardized approach to measure iron exposure and categorization of red and processed meat and other sources of organic and inorganic iron. This, together with the maintenance and expansion of food composition databases, with particular reference to iron content, would improve the quality of dietary assessments of iron intake for studies relating to iron and chronic disease. Assessments of systemic iron depots in such studies should be based on measurement of serum ferritin concentration.
- Iron intakes and iron status of vulnerable groups, particularly minority ethnic groups and infants aged up to 18 months, need to be better characterized.
- An improved understanding is required of the factors underlying differences in risk of iron deficiency anemia between women of reproductive age from low income populations and those in the general population.
- The extent to which foods fortified with iron, e.g., cereals and cereal products, and breast milk substitutes contribute to the supply of absorbed iron and to achieving adequate iron status, particularly in vulnerable groups, should be assessed.
- An improved understanding of the possible adverse effects of iron supplements in iron replete children is needed.
- Further randomized controlled trials with adequate power and sufficient duration are required to examine the effect of iron supplementation on mental development in children under three years old with iron deficiency anemia.
- Further studies are required on benefits, risks, and long-term effects of a delay in clamping the umbilical cord after birth until it has stopped pulsing.
